# An Approximation Framework for Solvers and Decision Procedures

**DOI:** 10.1007/s10817-016-9393-1

**Published:** 2016-11-10

**Authors:** Aleksandar Zeljić, Christoph M. Wintersteiger, Philipp Rümmer

**Affiliations:** 1 Uppsala University, Uppsala, Sweden; 2 Microsoft Research, Cambridge, UK

## Abstract

We consider the problem of automatically and efficiently computing models of constraints, in the presence of complex background theories such as floating-point arithmetic. Constructing models, or proving that a constraint is unsatisfiable, has various applications, for instance for automatic generation of test inputs. It is well-known that a naïve encoding of constraints into simpler theories (for instance, bit-vectors or propositional logic) often leads to a drastic increase in size, or that it is unsatisfactory in terms of the resulting space and runtime demands. We define a framework for systematic application of approximations in order to improve performance. Our method is more general than previous techniques in the sense that approximations that are neither under- nor over-approximations can be used, and it shows promising performance on practically relevant benchmark problems.

## Introduction

The construction of satisfying assignments (or, more generally, models) for a set of given constraints, or showing that no such assignments exist, is one of the most central problems in automated reasoning. Although the problem has been addressed extensively in research fields including constraint programming and more recently in satisfiability modulo theories (SMT), there are still constraint languages and background theories where effective model construction is challenging. Such theories are, in particular, arithmetic domains such as bit-vectors, nonlinear real arithmetic (or real-closed fields), and floating-point arithmetic (FPA); even when decidable, the high computational complexity of such languages turns model construction into a bottleneck in applications such as bounded model checking, white-box test case generation, analysis of hybrid systems, and mathematical reasoning in general.

We follow a recent line of research that applies the concept of *abstraction* to model construction (e.g., [3, 5, 10, 19]). In this setting, constraints are usually simplified prior to solving to obtain over- or under-approximations, or some combination thereof (*mixed abstractions*); experiments have shown that this concept can speed up model construction significantly. However, previous work in this area suffers from the fact that the definition of good over- and under-approximations is difficult and limiting, for instance in the context of floating-point arithmetic. We argue that the focus on over- and under-approximations is neither necessary nor optimal: as a more flexible alternative, we present a general algorithm that is able to incorporate *any form of approximation* in the solving process, including approximations that cannot naturally be represented as a combination of over- and under-approximations. Our method preserves essential properties like soundness, completeness, and termination.

For the purpose of empirical evaluation, we instantiate our procedure for the domain of floating-point arithmetic, and present an evaluation based on an implementation thereof within the Z3 theorem prover [22]. Experiments on practically relevant and satisfiable floating-point benchmark problems (SMT-LIB QF_FP) show an average speed-up of roughly one order of magnitude when compared to the naïve bit-blasting-based default decision procedure that comes with Z3. Further experiments show that the performance of our prototype implementation is also competitive with other state-of-the-art solvers for floating-point arithmetic.

While mainly intended for model generation, our method can also show unsatisfiability of constraints, and thanks to a new technique for refinement of unsatisfiable (sub-)problems, only a small performance penalty is incurred on them. However, we believe that further research is necessary to improve reasoning for unsatisfiable problems, even though our current prototype implementation exhibits satisfactory performance on unsatisfiable benchmark problems.

The contributions of this article are as follows:

1. a general method for model construction that can make use of arbitrary approximations of constraints,
2. an instantiation of our method for the theory of floating-point arithmetic,
3. refinement techniques for approximate models and unsatisfiable problems, as well as
4. an experimental evaluation of a prototype implementation of all proposed methods.

### Motivating Example

To illustrate our motivation and the resulting techniques, consider a heavily simplified software proportional-integral (PI) controller operating on floating-point data, as shown in Algorithm 1.
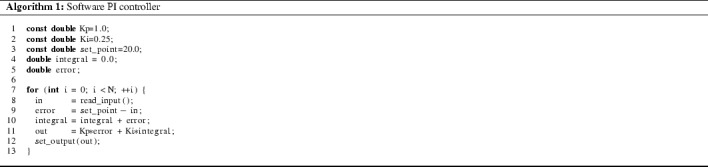


All variables in this example range over double precision (64-bit) IEEE-754 floating-point numbers. The controller is initialized with the set_point value and the constants Kp and Ki, it reads input values (in; e.g., from a sensor) via function read_input, and it computes output values (out) which control the system through the function set_output. The controller computes the control values in such a way, that the input values are as close to set_point as possible. For simplicity, we assume that there is a bounded number N of control iterations.

Suppose we want to prove that if the input values stay within the range $18.0 \le \textsf {in} \le 22.0$, then the control values will stay within a range that we consider safe, for instance $-3.0 \le \textsf {out} \le +3.0$. This property is true of our controller only for two control iterations, but it can be violated within three.

A bounded model checking approach to this problem produces a series of formulas, one for each $\mathsf {N}$ and it then checks the satisfiability of those formulas (usually in sequence). Today, most (precise) solvers for floating-point formulas implement this satisfiability check by means of *bit-blasting,* i.e., using a bit-precise encoding of FPA semantics as a propositional formula. Due to the complexity of FPA, the resulting formulas grow very quickly, and tend to overwhelm even the fastest SAT/SMT solvers. For example, an unrolling of the PI controller example to N = 100 steps cannot be solved by Z3 within an hour of runtime (see Table 1).

**Table 1:** Behavior of Z3 on the PI controller example

Bound N	1	2	5	10	20	30	40	50	100
Clauses ($\times 10^3$)	96	230	630	1298	2633	3969	5304	6639	13316
Variables ($\times 10^3$)	12	28	78	161	326	492	657	822	1649
Z3 time (s)	1	5	19	27	288	1190	1962	3297	>1h

However, this example has the property that the full range of floating-point numbers is not required to find suitable program inputs; essentially a prover just needs to find a sequence of inputs such that the errors add up to a sum that is greater than 3.0. There is no need to consider numbers with large magnitude, or a large number of significant digits/bits. We postulate that this situation is typical for many practical applications. Since bit-precise treatment of floating-point numbers is clearly wasteful in this setting, we might consider some of the following alternatives:

- all operations in the program can be evaluated in **real** instead of floating-point arithmetic. For problems with only linear operations, such as the program at hand, this enables the use of highly efficient solvers based on linear programming (LP). However, the straight-forward encoding into LP would ignore the possibility of overflows or rounding errors. A bounded model checking approach based thereupon will therefore be neither sound nor complete. Further, little is gained in terms of computational complexity for nonlinear constraints.
- operations can be evaluated in **fixed-point** arithmetic. Again, this encoding does not preserve the overflow- and rounding-semantics of FPA, but it enables solving using more efficient bit-vector encodings and solvers.
- operations can be evaluated in FPA with **reduced precision**: we can use single precision numbers, or other formats even smaller than that.

Strictly speaking, soundness and completeness are lost in all three cases, since the precise nature of overflows and rounding in FPA is ignored. All three methods enable, however, the efficient computation of *approximate models,* which are likely to be “close” to genuine double-precision FPA models, for some notion of closeness. In this paper, we define a general framework for model construction with approximations. In order to establish soundness and completeness of our model construction algorithm, the framework contains a *model reconstruction* phase, in which approximate models are translated into precise models. This reconstruction may fail, in which case *approximation refinement* is used to iteratively increase the precision of approximate models.

## Related Work

Related work to our contribution falls into two categories: general abstraction and approximation frameworks, and specific decision procedures for floating-point arithmetic.

The concept of abstraction (and approximation) is central to software engineering and program verification, and it is increasingly employed in general mathematical reasoning and in decision procedures. Usually, and in contrast to our work, only under- and over-approximations are considered, i.e., the formula that is solved either implies or is implied by an approximate formula (or abstraction). Counter-example guided abstraction refinement [7] is a general concept that is applied in many verification tools and decision procedures (e.g., even on a relatively low level like in QBF [18] or in model based quantifier instantiation for SMT [13]).

A general framework for abstracting decision procedures is Abstract CDCL, recently introduced by D’Silva et al. [10], which was also instantiated with great success for FPA [2, 11]. This approach relies on the definition of suitable abstract domains for constraint propagation and learning. In our experimental evaluation, we compare to the FPA decision procedure in MathSAT, which is an instance of ACDCL. ACDCL can also be integrated with our framework, e.g., to solve approximations. A further framework for abstraction in theorem proving was proposed by Giunchiglia et al. [14]. Again, this work focuses on under- and over-approximations, not on other forms of approximation.

Specific instantiations of abstraction schemes in related areas include the bit-vector abstractions by Bryant et al. [5] and Brummayer and Biere [4], as well as the (mixed) floating-point abstractions by Brillout et al. [3]. Van Khanh and Ogawa present over- and under-approximations for solving polynomials over reals [19]. Gao et al. [12] present a $\delta$-complete decision procedure for nonlinear reals, considering over-approximations of constraints by means of $\delta$-weakening.

There is a long history of formalization and analysis of FPA concerns using proof assistants, among others in Coq by Melquiond [21] and in HOL Light by Harrison [15]. Coq has also been integrated with a dedicated floating-point prover called Gappa by Boldo et al. [1], which is based on interval reasoning and forward error propagation to determine bounds on arithmetic expressions in programs [9]. The ASTRÉE static analyzer [8] features abstract interpretation-based analyses for FPA overflow and division-by-zero problems in ANSI-C programs. The SMT solvers MathSAT [6], Z3 [22], and Sonolar [20], all feature (bit-precise) conversions from FPA to bit-vector constraints.

## Preliminaries

We establish a formal basis in the context of multi-sorted first-order logic (e.g., [16]). A signature $\varSigma =(S, P, F, \alpha )$ consists of a set of sort symbols *S*, a set of sorted predicate symbols *P*, a set of sorted function symbols *F*, and a sort mapping $\alpha$. Each predicate and function symbol $g \in P \cup F$ is assigned a $(k+1)$-tuple $\alpha (g)$ of argument sorts (with $k\ge 0$), where *k* is the arity of the symbol. Constants are considered to be nullary function symbols. Also, the Boolean sort symbol is included in the set of sorts, i.e. $s_b\in S$. We assume a countably infinite set *X* of variables, and (by abuse of notation) overload $\alpha$ to assign sorts also to variables. Given a multi-sorted signature $\varSigma$ and variables *X*, the notions of well-sorted terms, atoms, literals, clauses, and formulas are defined as usual. The function $fv (\phi )$ denotes the set of free variables in a formula $\phi$. In what follows, we assume that all formulas are quantifier-free.

A $\varSigma$-structure $m=(U,I)$ with underlying universe *U* and interpretation function *I* maps each sort $s \in S$ to a non-empty set $I(s) \subseteq U$, each predicate $p \in P$ of sorts $(s_1,s_2,\ldots ,s_k)$ to a relation $I(p) \subseteq I(s_{1})\times I(s_{2}) \times \ldots \times I(s_{k})$, and each function $f \in F$ of sort $(s_1,s_2,\ldots ,s_k,s_{k+1})$ to a set-theoretic function $I(f) : I(s_{1}) \times I(s_{2}) \times \ldots \times I(s_{k}) \rightarrow I(s_{k+1})$. A variable assignment $\beta$ under a $\varSigma$-structure *m* maps each variable $x\in X$ to an element $\beta (x) \in I(\alpha (x))$. The valuation function $val _{m,\beta }(\cdot )$ is defined for terms and formulas in the usual way. A theory *T* is a pair $(\varSigma , M)$ of a multi-sorted signature $\varSigma$ and a class of $\varSigma$-structures *M*. A formula $\phi$ is *T*-satisfiable if there is a structure $m \in M$ and a variable assignment $\beta$ such that $\phi$ evaluates to $true$; we denote this by $m, \beta \models _T \phi$, and call $\beta$ a *T*-solution of $\phi$.

## The Approximation Framework

**Fig. 1:**
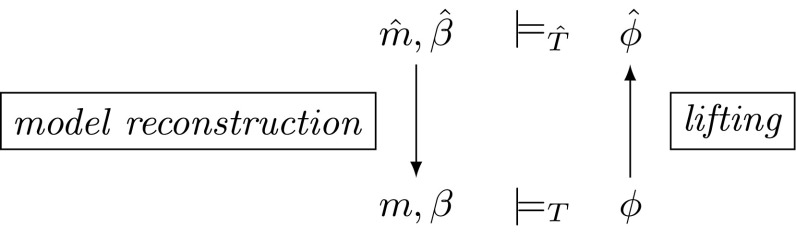
Commutativity graph showing how the model $m,\beta$ can be obtained via approximation theory $\hat{T}$

We describe a model construction procedure for formulas $\phi$ over a set of variables *X* in a theory *T*. The goal is to obtain a *T*-solution of $\phi$. The main idea underlying our method is to replace the theory *T* with an *approximation theory* $\hat{T}$, which enables explicit control over the precision used to evaluate theory operations. In our method, the *T*-problem $\phi$ is first lifted to a $\hat{T}$-problem $\hat{\phi }$, then solved in the theory $\hat{T}$, and finally, if a solution is found, it is translated back to a *T*-solution. The benefit of using the theory $\hat{T}$ is that different levels of approximation may be used during computation. We will use the theory of floating-point arithmetic as a running example for instantiation of this framework (Fig. 1).

### Approximation Theories

In order to formalize the approach of finding models by means of approximation, we construct the *approximation theory* $\hat{T} = (\hat{\varSigma },\hat{M})$ from *T*, by extending all function and predicate symbols with a new argument representing the *precision* to which the function or predicate should be computed.

*Syntax* We introduce a new sort for the precision $s_p$, and a new predicate symbol $\preceq$ which orders precision values. The signature ${\hat{\varSigma } = (\hat{S}, \hat{P}, \hat{F}, \hat{\alpha })}$ is obtained from $\varSigma$ in the following manner: $\hat{S} = S \cup \{s_{p}\}$; the set of predicate symbols is extended with the new predicate symbol $\preceq , \hat{P} = P \cup \{\preceq \}$; the set of function symbols is extended with the new constant $\omega$, representing the maximum precision value, $\hat{F} = F \cup \{\omega \}$; the sort function $\hat{\alpha }$ is defined as

$$\begin{aligned} \hat{\alpha }(g) = {\left\{ \begin{array}{ll} (s_p,s_1,s_2,\ldots ,s_n) &{} \text {if}~ g \in P\cup F~ \text {and}~ \alpha (g)= (s_1,s_2 ,\ldots , s_n) \\ (s_p, s_p, s_b) &{} \text {if}~ g = \;\preceq \\ (s_p) &{} \text {if}~ g = \omega \\ \alpha (g)\ &{} \text {otherwise} \end{array}\right. } \end{aligned}$$

Note that constant symbols become unary function symbols instead.

*Semantics* $\hat{\varSigma }$-structures $(\hat{U},\hat{I})$ enrich the original $\varSigma$-structures by providing approximate versions of function and predicate symbols. The resulting operations may be under- or over-approximations, but they may also be approximations that are close to the original operations’ semantics by some other metric. The degree of approximation is controlled with the help of the precision argument. We assume that the set $\hat{M}$ of $\hat{\varSigma }$-structures satisfies the following properties:

- for every structure $(\hat{U},\hat{I}) \in \hat{M}$, the relation $\hat{I}(\preceq )$ is a partial order on $\hat{I}(s_{p})$ that satisfies the ascending chain condition (every ascending chain is finite), and that has the unique greatest element $\hat{I}(\omega ) \in \hat{I}(s_{p})$;
- for every structure $(U, I) \in M$, an approximation structure $(\hat{U},\hat{I}) \in \hat{M}$ extending (*U*, *I*) exists, together with an embedding $h:U \mapsto \hat{U}$ such that, for every sort $s \in S$, function $f \in F$, and predicate $p \in P$:
  $$\begin{aligned} h(I(s))&~~\,\subseteq ~~ \hat{I}(s)\\ (a_1, \ldots , a_n) \in I(p)&\iff (\hat{I}(\omega ), h(a_1), \ldots , h(a_n)) \in \hat{I}(p)&(a_i \in I(\alpha (p)_i))\\ h(I(f)(a_1, \ldots , a_n))&~~\,=~~ \hat{I}(f)(\hat{I}(\omega ), h(a_1), \ldots , h(a_n))&(a_i \in I(\alpha (f)_i)) \end{aligned}$$
- vice versa; for every approximation structure $(\hat{U},\hat{I}) \in \hat{M}$ there is a structure $(U, I) \in M$ that is similarly embedded in $(\hat{U},\hat{I})$.

These properties ensure that every *T*-model has a corresponding $\hat{T}$-model, i.e. that no models are lost. Interpretations of function and predicate symbols under $\hat{I}$ with maximal precision are isomorphic to their original interpretation under *I*. The interpretation $\hat{I}$ should interpret the function and predicate symbols in such a way that their interpretations for a given value of the precision argument approximate the interpretations of the corresponding function and predicate symbols under *I*. And finally, that it is possible to translate every $\hat{T}$-model into some *T*-model, using a mapping $h^{-1}$ that reverses the embedding *h* (not necessarily its mathematical inverse, since *h* is rarely going to be bijective, but an inverse in spirit).

### Application to Floating-Point Arithmetic

The IEEE-754 standard for floating-point numbers [17] defines floating-point numbers, their representation in bit-vectors, and the corresponding operations. Most crucially, bit-vectors of various sizes are used to represent the significant and the exponent of numbers; e.g., double-precision floating-point numbers are represented by using 11 bits for the exponent and 53 bits for the significant. denote the subset of reals that can be represented as floating-point numbers *s* significant bits and *e* exponent bits by $$ FP _{s,e}$$:

$$\begin{aligned} FP _{s,e} = \left\{ \begin{array}{ll} &{} sgn \in \{0,1\},\\ (-1)^{ sgn }\cdot sig \cdot 2^{ exp - s } \mid &{} sig \in \{0, \ldots , 2^{s}-1\},\\ &{} exp \in \{ -2^{e-1} + 3, \ldots , 2^{e-1} \} \end{array} \right\} \cup \left\{ \begin{matrix} NaN , &{} +\infty , \\ -\infty , &{} -0 \\ \end{matrix} \right\} \end{aligned}$$

The set consists of: 1. normalized numbers (in practice encoded with an implicit leading bit set to 1), 2. subnormal numbers, and 3. special values. The definition does not discriminate between normal and subnormal numbers and any value with multiple representations loses the multiplicity in the set. Since the reals do not contain a signed zero value it is included explicitly with the other special values.

#### Proposition 1

(Inclusion property) FP domains grow monotonically when increasing *e* or *s*, i.e., $FP _{s',e'} \subseteq FP _{s,e}$ provided that $s' \le s$ and $e' \le e$; we call this the *inclusion property*.

For fixed values *e* of exponent bits and *s* of significant bits, FPA can be modeled as a theory in our sense. We denote this theory by $$ TF _{s,e}$$, and write $$s_{f}$$ for the sort of FP numbers, and $$s_{r}$$ for the sort of rounding modes. The various FP operations are represented as functions and predicates of the theory; for instance, floating-point addition turns into the function symbol $$\oplus $$ with signature $$\alpha (\oplus )=(s_{r}, s_{f},s_{f},s_{f})$$. Additional constants of sort $$s_{r}$$ are provided for the five rounding modes in the IEEE-754 standard, namely

- $$ RoundTowardZero $$,
- $$ RoundNearestTiesToEven $$,
- $$ RoundNearestTiesToAway $$,
- $$ RoundTowardPositive $$, and
- $$ RoundTowardNegative $$.

The semantics of $$ TF _{s,e}$$ is defined by a single structure $$(U_{s,e}, I_{s,e})$$ with $$I_{s,e}(s_{f})= FP _{s,e}$$. The semantics of floating-point operations is derived from the corresponding operations over reals, except in cases where the resulting values are not representable as floating-point numbers; then rounding takes place in accordance with the chosen rounding mode.

*FPA approximation theories* We construct the approximation theory $\hat{ TF }_{s,e}$, by introducing the precision sort $s_p$, predicate symbol $\preceq$, and a constant symbol $\omega$. The function and predicate symbols have their signature changed to include the precision argument. For example, the signature of the floating-point addition symbol $\oplus$ is $\hat{\alpha }(\oplus )=(s_p, s_{r}, s_{f}, s_{f}, s_{f})$ in the approximation theory.

The semantics of the approximation theory $\hat{ TF }_{s,e}$ is again defined through a singleton set $\hat{M}_{s,e}=\{ (\hat{U}_{s,e}, \hat{I}_{s,e})\}$ of structures. The universe of the approximation theory extends the original universe with a set of integers which are the domain of the precision sort, i.e., $\hat{U}_{s,e}= U_{s,e} \cup \{0,1, \ldots , n\}$, $\hat{I}_{s,e}(s_p)= \{0,1, \ldots , n\}$, and $\hat{I}_{s,e}(\omega ) = n$. The embedding *h* is the identity mapping. In order to use precision to regulate the semantics of FP operations, we introduce the notation $(s,e) \downarrow p$ to denote the number of bits in reduced precision $p \in \{0, 1, \ldots , n\}$; more specifically we define

$$\begin{aligned} (s,e) \downarrow p ~=~ \left( 3 + \left\lceil (s - 3) \cdot \frac{p}{n} \right\rceil ,\; 3 + \left\lceil (e - 3) \cdot \frac{p}{n} \right\rceil \right) , \end{aligned}$$

which scales the floating-point sort, however the smallest sort it scales to is $$ FP _{3,3}$$ since smaller well-defined domains contain mostly special values. The approximate semantics of functions is derived from the FP semantics for the reduced bit-widths. For example, $$\oplus $$ in approximation theory $$\hat{ TF }_{s,e}$$ is defined as

$$\begin{aligned} \hat{I}_{s,e}(\oplus )(p,r,a,b) ~=~ cast_{s,e}( I_{(s,e)\downarrow p}(\oplus ) (r, cast _{(s,e)\downarrow p}(a), cast _{(s,e)\downarrow p}(b))) \end{aligned}$$

This definition uses the function $cast_{s,e}$ to map any FP number to a number with *s* significant bits and *e* exponent bits, i.e., $cast_{s,e}(a) \in FP _{s,e}$ for any $a \in FP _{s',e'}$. If $s \ge s'$ and $e \ge e'$ then the casting function does not change the value of the argument, only its sort, i.e., $cast_{s,e}(a) = a$. Otherwise, the cast function performs rounding (if necessary) using a fixed rounding mode. Note that many occurrences of $cast_{s,e}$ can be eliminated in practice, if they only concern intermediate results. For example, consider $\oplus (c_1,\otimes (c_2,a_1,a_2),a_3)$. The result of $\otimes (c_2,a_1,a_2)$ can be directly cast to precision $c_1$ without the need of casting up to full precision when calculating the value of the expression.

### Lifting Constraints to Approximate Constraints

In order to solve a constraint $\phi$ using an approximation theory $\hat{T}$, it is first necessary to lift $\phi$ to an extended constraint $\hat{\phi }$ that includes explicit variables $c_l$ for the precision of each operation. This is done by means of a simple traversal of $\phi$, using a recursive function *L* that receives a formula (or term) $\phi$ and a position $l \in \mathbb {N}^*$ as argument. For every position *l*, the symbol $c_l$ denotes a fresh variable of the precision sort $\alpha (c_l) = s_p$ and we define

$$\begin{aligned} L(l, \lnot \phi )&~=~ \lnot L(l.1, \phi )\\ L(l, \phi \circ \psi )&~=~ L(l.1, \phi ) \circ L(l.2, \psi )&(\circ \in \{\vee , \wedge \})\\ L(l, x)&~=~ x&(x \in X)\\ L(l, g(t_1, \ldots , t_n))&~=~ g(c_l, L(l.1, t_1), \ldots , L(l.n, t_n))&(g \in F \cup P) \end{aligned}$$

Then we obtain the lifted formula $\hat{\phi } = L(\epsilon , \phi )$, where $\epsilon$ denotes an empty word. Since *T*-structures can be embedded into $\hat{T}$-structures, it is clear that no models are lost as a result of lifting:

#### Lemma 1

(Completeness) If a *T*-constraint $\phi$ is *T*-satisfiable, then the lifted constraint $\hat{\phi } = L(\epsilon , \phi )$ is $\hat{T}$-satisfiable as well.

In practice, the lifting can make use of expression sharing and cache lifted terms to avoid introduction of unnecessary precision variables or redundant sub-terms.

An approximate model that chooses full precision for all operations induces a model for the original constraint:

#### Lemma 2

(Fully precise operations) Let $\hat{m} = (\hat{U}, \hat{I})$ be a $\hat{T}$-structure, and $\hat{\beta }$ a variable assignment. If $\hat{m}, \hat{\beta } \models _{\hat{T}} \hat{\phi }$ for an approximate constraint $\hat{\phi } = L(\epsilon , \phi )$, then $m, \beta \models _T \phi$, provided that: 1. there is a *T*-structure *m* embedded in $\hat{m}$ via *h*, and a variable assignment $\beta$ such that $h(\beta (x)) = \hat{\beta }(x)$ for all variables $x \in fv (\phi )$, and 2. $\hat{\beta } (c_l) = \hat{I}(\omega )$ for all precision variables $c_l$ introduced by *L*.

The fully precise case however, is not the only case in which an approximate model is easily translated to a precise model. For instance, approximate operations might still yield a precise result for some arguments. Examples of this are constraints in floating-point arithmetic with small integer or fixed-point arithmetic solutions.

A variation of Lemma 2 is obtained by not requiring that all operations are at maximum precision, but that each operation is at a sufficiently high precision, such that it evaluates to the same value as the maximally precise operation in all relevant cases:

#### Lemma 3

(Locally precise operations) Suppose $\hat{m}, \hat{\beta } \models _{\hat{T}} \hat{\phi }$ for an approximate constraint $\hat{\phi } = L(\epsilon , \phi )$, such that: 1. there is a *T*-structure *m* embedded in $\hat{m}$ via *h* and a variable assignment $\beta$ such that $h(\beta (x)) = \hat{\beta }(x)$ for all variables $x \in fv (\phi )$, and 2. for every sub-expression $g(c_l, \bar{t})$ with $g \in F \cup P$, it holds that $val _{\hat{m}, \hat{\beta }}(g(c_l, \bar{t})) = val _{\hat{m}, \hat{\beta }}(g(\omega , \bar{t}))$. Then $m, \beta \models _T \phi$.

*Applied to FPA*Because floating-point numbers of varying bit-widths enjoy the inclusion property, it is easy to see that an approximate model $\hat{m}, \hat{\beta }$ for an approximate $\hat{\phi }$ which, during model evaluation (validation) does not trigger any rounding decisions, must equally entail the original, precise constraint $\phi$.

#### Theorem 1

(Exact evaluation) Let $\hat{m}$ be the unique element of the singleton set of structures $\hat{m}_{s,e}$ of theory $\hat{TF}_{s,e}$. Suppose ${\hat{m}, \hat{\beta } \models _{\hat{TF}_{s,e}} \hat{\phi }}$ for an approximate constraint $\hat{\phi } = L(\epsilon , \phi )$, such that: 1. *m* is the *T*-structure of theory $TF_{s,e}$ embedded in $\hat{m}$ via *h* (which is the identity function) and $\beta$ a variable assignment such that $h(\beta (x)) = \hat{\beta }(x)$ for all variables $x \in fv (\phi )$, and 2. it is possible to evaluate all operations $\hat{\phi }$ exactly, i.e. without rounding. Then $m, \beta \models _{TF_{s,e}} \phi$.

#### Proof

By Lemma 3 and the inclusion property. $\square$

#### Example 1

Lifting the constraints. Consider again the PI controller example given in Sect. 1.1. Suppose that the program loop is unrolled *N* times and translated into single static assignment form, resulting in a set of equations that can be checked for satisfiability. Variables corresponding to the values of program variables at the end of each loop iteration are used as inputs for the next iteration. For the first loop iteration, this leads to the following constraint:where $Kp , Ki$, and $set\_point$ are constant (set to the values given in the PI program, in equations not shown here), and the constant $rm$ stores the rounding mode. The negated output condition encodes the fact that we search for a violation of the property in any loop iteration.

After lifting those constraints, we obtain the following formula:The variables $p_0, p_1, \ldots , p_8, \ldots$ are freshly introduced precision variables of the sort $s_p$. We use the notation $\oplus _{ rm }^p$ to express that $\oplus$ is an operator with four arguments: the precision $p_2$, the rounding mode $rm$, and the two numbers to be added; and similarly for the other operators.

## Model Refinement Scheme

In the following sections, we will use the approximation framework to successively construct more and more precise solutions of given constraints, until eventually either a genuine solution is found, or the constraints are determined to be unsatisfiable. We fix a partially ordered precision domain $(D_p, \preceq _p)$ (where, as before, $\preceq _p$ satisfies the ascending chain condition, and has a greatest element), and consider approximation structures ${(\hat{U}, \hat{I})}$ such that ${\hat{I}(s_{p})} = D_p$ and $\hat{I}(\preceq ) = \;\preceq _p$.

Given a lifted constraint $\hat{\phi } = L(\epsilon , \phi )$, let $X_p \subseteq X$ be the set of precision variables introduced by the function *L*. A *precision assignment* $\gamma : X_p \rightarrow D_p$ maps the precision variables to precision values. We write $\gamma \preceq _p \gamma '$ if for all variables $c_l \in X_p$ we have $\gamma (c_l) \preceq _p \gamma '(c_l)$. Precision assignments are partially ordered by $\preceq _p$. There is a greatest precision assignment $\gamma _\omega$, which maps each precision variable to $\omega$. The precision assignment can be obtained from the variable assignment $\hat{\beta }$ after the solving, but due to its role in controlling the search through the space of approximations (by fixing its values before solving) we separate it from $\beta$.

**Fig. 2:**
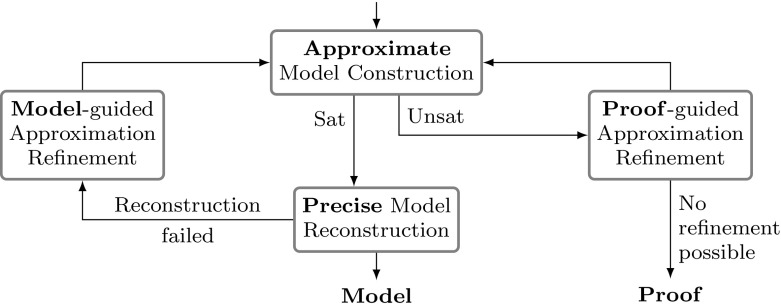
The model construction process

The proposed procedure is outlined in Fig. 2. First, an initial precision assignment $\gamma$ is chosen, depending on the theory *T*. In *Approximate Model Construction,* the procedure tries to find $(\hat{m}, \hat{\beta })$, a model of the approximated constraint $\hat{\phi }$. If $(\hat{m}, \hat{\beta })$ is found, *Precise Model Reconstruction* tries to translate it to $(m,\beta )$, a model of the original constraint $\phi$. If this succeeds, the procedure stops and returns the model. Otherwise, *Model-guided Approximation Refinement* uses $(m, \beta )$ and $(\hat{m}, \hat{\beta })$ to increase the precision assignment $\gamma$. If Approximate Model Construction cannot find any model $(\hat{m}, \hat{\beta })$, then *Proof-guided Approximation Refinement* decides how to modify the precision assignment $\gamma$. If the precision assignment is maximal and cannot be further increased, the procedure has determined unsatisfiability. In the following sections we provide additional details for each of the components of our procedure.

*General properties* Since $\preceq _p$ has the ascending chain property, our procedure is guaranteed to terminate and either produce a genuine precise model, or detect unsatisfiability of the constraints. The potential benefits of this approach are that it often takes less time to solve multiple smaller (approximate) problems than to solve the full problem straight away. The candidate models provide useful hints for the following iterations. The downside is that it might be necessary to solve the whole problem eventually anyway, which can be the case for unsatisfiable problems. Whether that is the case depends on the strategy used in the proof-guided approximation refinement, e.g., maximizing the precision of terms involved in an unsatisfiable core can cut down the overhead significantly compared to even increase in precision of all terms. Therefore, our approach is definitely useful when the goal is to obtain a model, e.g., when searching for counter-examples, but it can also perform well on unsatisfiable formulas, e.g., when a small unsatisfiable core can be discovered quickly.

### Approximate Model Construction

Once a precision assignment $\gamma$ has been fixed, existing solvers for the operations in the approximation theory can be used to construct a model $\hat{m}$ and a variable assignment $\hat{\beta }$ s.t. $\hat{m},\hat{\beta }\models _{\hat{T}}\hat{\phi }$. It is necessary that $\hat{\beta }$ and $\gamma$ agree on $X_p$. As an optimization, the model search can be formulated in various theory-dependent ways that provide a heuristic benefit to Precise Model Reconstruction. For example, the search can prefer models with small values of some error criterion, or to attempt to find models that are similar to models found in earlier iterations. This can be done by encoding the problem as an optimization query, assuming one can encode the desired criteria as part of the formula.

*Applied to FPA* Since our FP approximations are again formulated using FP semantics, any solver for FPA can be used for Approximate Model Construction. In our implementation, the lifted constraints $\hat{\phi }$ of $\hat{TF}_{s,e}$ are encoded in bit-vector arithmetic, and then bit-blasted and solved using a SAT solver. The encoding of a particular function or predicate symbol uses the precision argument to determine the floating-point domain of the interpretation. This kind of approximation reduces the size of the encoding of each operation, and results in smaller problems handed over to the SAT solver. An example of theory-specific optimization of the model search is to prefer models where no rounding occurs during evaluation.

### Reconstructing Precise Models

Algorithm 2 provides a high-level sketch for the model reconstruction phase. This algorithm attempts to produce a model $(m,\beta )$ for the original formula $\phi$ from an approximate model $(\hat{m}, \hat{\beta })$ obtained by solving ${\hat{\phi }}$. Since we consider arbitrary approximations (which might be neither over- nor under-), this translation is non-trivial; for instance, approximate and precise operations might exhibit different rounding behavior. In practice, it might still be possible to ‘patch’ approximate models that are close to real models, avoiding further refinement iterations.
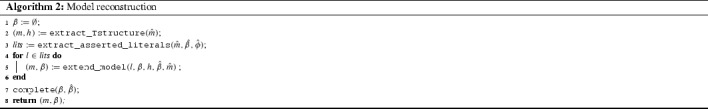


Note that by definition it is possible to embed a *T*-structure *m* in $\hat{m}$. It is retrieved, together with the embedding *h*, by extract_Tstructure in Algorithm 2. The structure *m* and *h* will be used to evaluate $\phi$ using values from $\hat{\beta }$. The function extract_asserted_literals determines a set $lits$ of literals in $\hat{\phi }$ that are true under $(\hat{m}, \hat{\beta })$, such that the conjunction $\bigwedge lits$ implies $\hat{\phi }$. For instance, if $\hat{\phi }$ is in CNF, one literal per clause can be selected that is true under $(\hat{m}, \hat{\beta })$. Any pair $(m, \beta )$ that satisfies the literals in $lits$ will be a *T*-model of $\phi$.

The procedure then iterates over $lits$, and successively constructs a valuation $\beta : X \rightarrow U$ such that $(m, \beta )$ satisfies all selected literals, and therefore is a model of $\phi$ (extend_model). During this loop, we assume that $\beta$ is a *partial* valuation defined only for some of the variables in *X*. We use the notation $\beta \uparrow h$ to lift $\beta$ from *m* to $\hat{m}$, setting all precision variables to greatest precision; formally defined as

$$\begin{aligned} (\beta \uparrow h)(x) ~=~ {\left\{ \begin{array}{ll} \hat{I}(\omega ) &{} \text {if}~ x \in X_p\\ h(\beta (x)) &{} \text {otherwise.} \end{array}\right. } \end{aligned}$$

The precise implementation of extend_model is theory-specific. In general, the function first attempts to evaluate a literal *l* as $val _{\hat{m}, \beta \uparrow h}(l)$. If this fails, the valuation $\beta$ has to be extended, for instance by including values ${\hat{\beta }(x)}$ for variables *x* not yet assigned in $\beta$.

After all literals have been successfully asserted, $\beta$ may be incomplete, so we complete it (either randomly or by mapping value assignments from $\hat{\beta }$) and return the model $(m,\beta )$. Note that, if all the asserted literals already have maximum precision assigned then, by Lemma 2, model reconstruction cannot fail.

*Applied to FPA*The function extract_Tstructure is trivial for our FPA approximations, since *m* and $\hat{m}$ coincide for the sort $s_{f}$ of FP numbers. Further, by approximating FPA using smaller domains of FP numbers, all of which are subsets of the original domain, reconstruction of models is easy in some cases and boils down to padding the obtained values with zero bits. The more difficult cases concern literals with rounding in approximate FP semantics, since a significant error emerges when the literal is re-interpreted using higher-precision FP numbers. A useful optimization is special treatment of equalities $x=t$ in which one side is a variable *x* not assigned in $\beta$, and all right-hand side variables are assigned. In this case, the choice $\beta (x) := val _{\hat{m}, \beta \uparrow h}(t)$ will satisfy the equation. Use of this heuristic partly mitigates the negative impact of rounding in approximate FP semantics, since the errors originating in the $(\hat{m}, \hat{\beta })$ will not be present in $(m,\beta )$. The heuristic is not specific to the floating-point theory, and can be carried over to other theories as well.

#### Example 2

— Model reconstruction. In order to illustrate how precise model reconstruction works, recall the formula obtained in Example 1. We fix the number of PI controller loop iterations to $$N = 1$$, but for reasons of presentation slightly change the values of the constants to $$ Ki = 0.125$$, $$ Kp = 1.25$$, and $$ set\_point = 3.0$$. Suppose further that the rounding mode is set to $$ RoundTowardZero $$, and that the property to be checked is the following: if $$ 2.0 \le in_{o} \le 4.0$$ then $$-1.0 \le out_{1} \le 1.0$$. Approximate model construction is performed with the precision assignment $$\gamma $$ that maps all precision variables $$p_0, p_1, \ldots , p_8$$ to 0, i.e., all computations are performed in the smallest floating-point domain $$ FP _{3,3}$$.

The columns in Table 2 represent, respectively, the variables in the formula, the terms those variables are assigned, their value in the model of the approximation $$\hat{\beta }$$ and their value in the reconstructed model $$\beta $$ . The variables in the table are topologically sorted, i.e., their order corresponds to the order of computation in the program, which allows propagation of the rounding error through the formula by interpreting equality as assignment when possible. Before proceeding to model reconstruction, the reader should note that evaluation under the given model $$\hat{\beta }$$ occurs without rounding, except for the value of $$ out _1$$, almost meeting the conditions of Lemma 3 and Theorem 1. The exact value of $$ out _1$$ cannot be represented in $$ FP _{3,3}$$ because $$1.375 = 1.011 \times 2^{0}$$ which requires 4 significant bits. Since there are only 3 significant bits available, the value is rounded according to the rounding mode $$ rm $$ (bold in Table 2). The given model indeed violates the desired property under $$I_{3,3}$$. The procedure constructs the model $$\beta $$, by evaluating the expressions using the interpretation function $$I_{53,11}$$. Initially, there are no values in $$\beta $$, so it is populated with values of variables that depend only on constants, cast up to the sort $$ FP _{53,11}$$. Next it proceeds to variables whose value depends on other variables. Since the order is topological, when there are no cycles (like in this example) all the values needed for evaluation are already available in $$\beta $$. The missing values in $$\beta $$ are computed by reevaluating the terms assigned to each variable using values of variables already in $$\beta $$. Since all the variables except $$out_1$$ are exact (in the sense that no rounding occurred), then by Lemma 3, their values in $$\beta $$ and $$\hat{\beta }$$ are (numerically) equal. In the case of $$out_1$$, however, there is a discrepancy between the two values. As there are no cyclic dependencies we can use the more precise value obtained using $$I_{53,11}$$. In general, the constructed model $$\beta $$ has to be checked against the constraints, because reconstruction is not guaranteed to succeed. In this example however, the reconstructed $$\beta $$ is indeed a satisfying assignment for the formula in question.

**Table 2:** Model reconstruction from $$ FP _{3,3}$$ to $$ FP _{53,11}$$

Variable	Defining term	$\hat{\beta }(x)$	$\beta (x)$
$Kp$	1.25	1.25	1.25
$Ki$	0.125	0.125	0.125
$set\_point$	3.0	3.0	3.0
$in_0$		4.0	4.0
$$ error _1$$	$$ set\_point \ominus _{ rm } in _0$$	1.0	1.0
$$ integral _1$$	$$ integral _{ init } \oplus _{ rm } error _1$$	1.0	1.0
$$ aux _a$$	$$ Kp \odot _{ rm } error _1$$	1.25	1.25
$$ aux _b$$	$$ Ki \odot _{ rm } error _1$$	0.125	0.125
$$ out _1$$	$$ aux _a \oplus _{ rm } aux _b$$	**1**.**25**	1.375

### Approximation Refinement

The overall goal of the refinement scheme outlined in Fig. 2 is to find a model of the original constraints using a series of approximations defined by precision assignments $\gamma$. We usually want $\gamma$ to be as small as possible in the partial order of precision assignments, since approximations with lower precision can be solved more efficiently. During refinement, the precision assignment is adjusted so that the approximation of the problem in the next iteration is closer to full semantics. Intuitively, this increase in precision should be kept as small as possible, but as large as necessary. Note that two different refinement procedures are required, depending on whether an approximation is satisfiable or not. We refer to these procedures as Model- and Proof-guided Approximation Refinement, respectively.

#### Model-guided Approximation Refinement

If a model $(\hat{m}, \hat{\beta })$ of $\hat{\phi }$ is obtained together with a reconstructed model $(m,\beta )$ that does *not* satisfy $\phi$, we use the procedure described in Algorithm 3 for adjusting $\gamma$. Since the model reconstruction failed, there are literals in $\hat{\phi }$ which are critical for $(\hat{m}, \hat{\beta })$, in the sense that they are satisfied by $(\hat{m}, \hat{\beta })$ and required to satisfy ${\hat{\phi }}$, but are not satisfied by $(m,\beta )$. Such literals can be identified through evaluation with both ${(\hat{m}, \hat{\beta })}$ and $(m,\beta )$ (as part of Algorithm 3 via extract_critical_literals), and can then be traversed, evaluating each sub-term under both structures. If a term $g(c_l,\bar{t})$ is assigned different values in the two models, it witnesses discrepancies between precise and approximate semantics; in this case, an error is computed using the error function, mapping to some suitably defined error domain (e.g., the real numbers $\mathbb {R}$ for errors represented numerically). The computed errors are then used to select those operations whose precision argument $c_l$ should be assigned a higher value.

Depending on refinement criteria, the rank_terms function can be implemented in different ways. For example, terms can be ordered according to the absolute error which was calculated earlier; if there are too many terms to refine, only a certain number of them will be selected for refinement. An example of a more complex criterion follows:

*Error-based selection* aims at refining the terms introducing the greatest imprecision first. The absolute error of an expression is determined by the errors of its sub-terms, and the error introduced by approximation of the operation itself. By calculating the ratio between output and input error, refinement tries to select those operations that cause the biggest *increase* in error. If we assume that theory *T* is some numerical theory (i.e., it can be mapped to reals in a straightforward manner), then we can define the error function (in Algorithm 3) as absolute difference between its arguments. Then $\varDelta (c_l)$ represents the *absolute error* of the term $g(c_l,\bar{t})$. This allows us to define the *relative error* $\delta (c_l)$ of the term $g(c_l,\bar{t})$ as

$$\begin{aligned} \delta (c_l) = \frac{\varDelta (c_l)}{| val _{\hat{m},\beta \uparrow h}(g(\omega ,\bar{t}))|} \; . \end{aligned}$$

Similar measures can be defined for non-numeric theories.

Since a term can have multiple sub-terms, we calculate the average relative input error; alternatively, minimum or maximum input errors could be used. We obtain a function characterizing the increase in error caused by an operation by defining

$$\begin{aligned} errInc (c_l) = \frac{\delta (c_l)}{1+ \frac{1}{k}\varSigma _{i=1}^{k}\delta (c_{l.i})} \;, \end{aligned}$$

where $$g(c_l, \bar{t})$$ represents the term being ranked. The function rank_terms then selects terms $$g(c_l, \bar{t})$$ with maximum error increase $$ errInc (c_l)$$. 
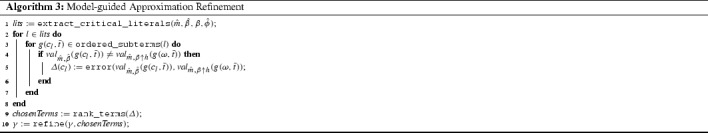


*Applied to FPA*The only difference to the general case is that we define relative error $\delta (c_l)$ to be $+\infty$ if a special value ($\pm \infty$, NaN) from $(\hat{m}, \hat{\beta })$ turns into a normal value under $(m,\beta )$. Our rank_terms function ignores terms which have an infinite average relative error of sub-terms. The refinement strategy will prioritize the terms which introduce the largest error, but in the case of special values it will refine the first imprecise terms that are encountered (in bottom up evaluation), because once the special values occur as input error to a term we have no way to estimate its actual error. After ranking the terms using the described criteria, rank_terms returns the top $30\%$ highest ranked terms. The precision of chosen terms is increased by a constant value.

#### Proof-Guided Approximation Refinement

When no approximate model can be found, some theory solvers may still provide valuable information why the problem could not be satisfied; for instance, proofs of unsatisfiability or unsatisfiable cores. While it may be (computationally) hard to determine which variables absolutely need to be refined in this case (and by how much), in many cases a loose estimate is easy to compute. For instance, a simple solution is to increase the precision of all variables appearing in the literals of an unsatisfiable core.

Given an unsatisfiable formula $\phi$ in conjunctive normal form (CNF), any unsatisfiable formula $\psi$ that is a conjunction of a subset of clauses in $\phi$ is called an unsatisfiable core. If a core $\psi$ has no proper subformula that is unsatisfiable, it is said to be a minimal unsatisfiable core. Given an unsatisfiable formula $\psi$ any formula $\phi$ that contains $\psi$ is also unsatisfiable, since $\psi$ is an unsatisfiable core of $\phi$ in that case. Generalizing this observation to our approximation theory $\hat{T}$ we get the following lemma:

##### Lemma 4

If $\psi$ is the unsatisfiable core of the lifted formula $\hat{\phi }$ under precision assignment $\gamma$ and all precision variables occurring in $\psi$ have maximal precision, i.e., $\gamma (x)=\omega$ for all $x \in X \cap vars (\psi )$, then formula $\phi$ is unsatisfiable.

The proof-guided refinement is shown in Algorithm 4. Lemma 4 provides a cheap stopping condition for proof-guided refinement. If the found core is at full precision (i.e., was obtained under the exact semantics), then regardless of precision of other constraints the original formula $\phi$ is guaranteed to be unsatisfiable. However, this is rarely the case (a number of refinement steps is necessary for precision variables to reach value $\omega$). Ideally the procedure would get a minimal core $\psi$ and it would be considerably smaller than the original constraint $\phi$. In that case, a satisfiability check of $\psi$ with all the terms at full precision (i.e., $\omega$) is likely to be easier than a satisfiability check of $\phi$. In the case the $\psi$ is an unsatisfiable core of $\phi$, this is discovered by solving a considerably smaller formula. If $\psi$ is not an unsatisfiable core of $\phi$, then its discovery is due to encoding at small precision, and once encoded at full precision, the search space is going to be expanded enough that the satisfiability check of $\psi$ is likely to be quick.

In the case that $\psi$ at full precision is an unsatisfiable core of $\phi$, proof-guided refinement returns UNSAT (by Lemma 4). Otherwise, we store the formula $\psi$ in seen_cores, to be able to skip the satisfiability check if we encounter it (or any of its subsets) in future iterations. All the precision variables are refined, since no useful information is hidden in the core.
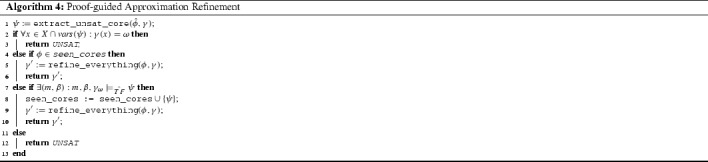


If the approximation theory uses a domain with the inclusion property and multiple iterations yield unsatisfiable approximations of the formula $\phi$ then the same solution space is explored repeatedly. Subsequent unsatisfiable iterations are undesirable due to the fact that every previous call is subsumed by the latest one, increasing the solving time unnecessarily. In the case when the approximation theory is FPA, this can be easily avoided by introducing blocking clauses. Between any two iterations, at least one variable had its precision increased, which means that after bit-blasting its encoding will contain additional variables. Since the domain satisfies the inclusion property, that means that all the newly introduced variables implicitly had value *false* in the previous iterations. If the approximation of the previous iteration was unsatisfiable, a single clause can be added to prevent revisiting that subspace. The blocking clause expresses that at least one of the newly introduced variables has to be *true* (i.e., non-zero).

*Example of blocking clauses.* Consider the following unsatisfiable formula:

$$\begin{aligned} x > y \wedge x / y < 1 \end{aligned}$$

Suppose that in the previous iteration *x* and *y* were approximated with fixed-point numbers with $m = 3$ integral and $f = 3$ fractional bits and that the approximation was unsatisfiable. After refinement, the next iteration will use $m=5$ and $f=5$ bits. Below the alignment of the two encodings by the decimal point is shown:

$$\begin{aligned} m_2m_1m_0&.f_0f_1f_2\\ m_4m_3m_2m_1m_0&.f_0f_1f_2f_3f_4\\ \end{aligned}$$

where $m_{i}$ denotes integral bits and $f_{i}$ fractional bits, for $i \in \{0,1,2,3,4,5\}$. In the previous iteration, the newly added bits $f_4,f_3,m_3,m_4$ implicitly had the value *false* (zero). Since the previous satisfiability check returned UNSAT, we can safely exclude those value combinations from the current search. In this example the blocking clause that should be added is

$$\begin{aligned} x_{f_4} \vee x_{f_3}\vee x_{m_3}\vee x_{m_4} \vee y_{f_4} \vee y_{f_3} \vee y_{m_3} \vee y_{m_4} \; . \end{aligned}$$

It evaluates to false when all the newly introduced bits have the values they implicitly had in the previous iteration, preventing further exploration of that part of the search subspace. This technique can be applied to any approximation theory with a domain that exhibits the inclusion property.

## Experimental Evaluation

To assess the efficacy of our method, we present results of an experimental evaluation obtained through an implementation of the approximation using smaller floating-point numbers (the ‘Smallfloat’ approximation) . We implemented this approach as a custom tactic [23] within the Z3 theorem prover [22]. All experiments were performed on Intel Xeon 2.5 GHz machines with a time limit of 1200 sec and a memory limit of 2 GB. The symbols  and  indicate that the time or the memory limit were exceeded.

*Implementation details.* For the sake of reproducibility of our experiments, we note that our implementation starts with an initial precision mapping $\gamma$ that limits the precision of all floating-point operations to $s = 3$ significant and $e = 3$ exponent bits. Upon refinement, operations receive an increase in precision that represents 20% of the width of the full precision. We do not currently implement any sophisticated proof-guided approximation refinement, but our prototype does feature core-based refinement as described in Sect. 5.3.2 and Algorithm 4.

*Evaluation.* Our benchmarks are taken from a recent evaluation of the ACDCL-based MathSAT, by Brain et al. [2]. This benchmark set contains 214 benchmarks, both satisfiable and unsatisfiable ones. The benchmarks originate from verification problems of C programs performing numerical computations, where ranges and error bounds of variables and expressions are verified; other benchmarks are randomly generated systems of inequalities over bounded floating-point variables. We evaluate two versions of our implementation of Smallfloat approximation, one with a simple proof-guided refinement denoted Smallfloat (no cores) and the other featuring core-based proof-guided refinement denoted Smallfloat. We compare against Z3 [22] and MathSAT [6].

**Table 3:** Evaluation statistics

	Z3 (Default)	MathSAT (Default)	MathSAT (ACDCL)	Smallfloat (no cores)	Smallfloat (Default)
Satisfiable	86	**95**	77	91	92
Unsatisfiable	59	67	**76**	53	64
Total	145	**162**	153	144	159

Bold values denote the solver with most instances solved

**Fig. 3:**
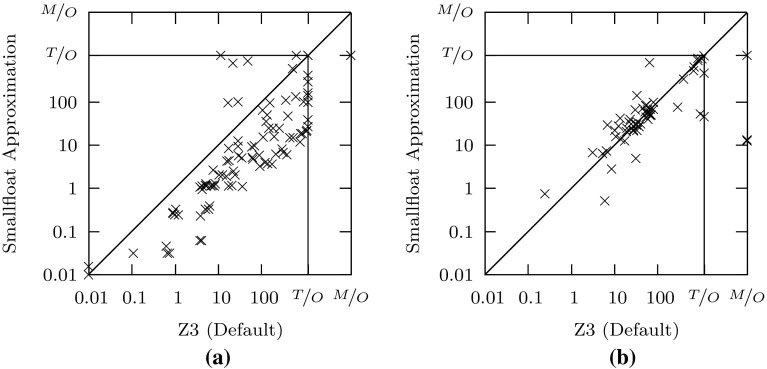
Comparisons of our method with the bit-blasting-based decision procedure in Z3. **a** Satisfiable. **b** Unsatisfiable

**Fig. 4:**
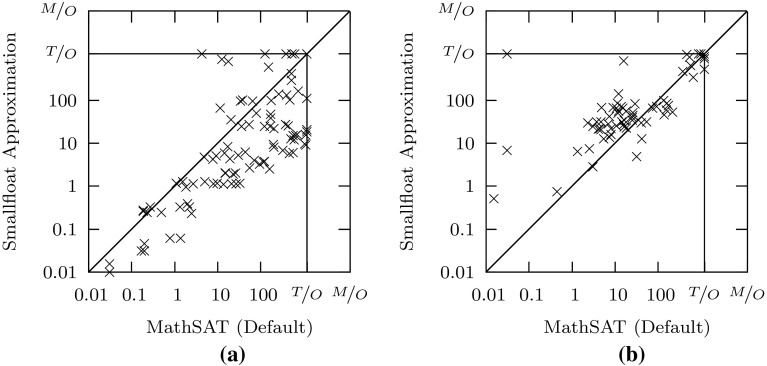
Comparison of our approximation method with the bit-blasting-based decision procedure in MathSAT. **a** Satisfiable. **b** Unsatisfiable

The results we obtain are briefly summarized in Table 3, which shows that our method solves more (satisfiable and unsatisfiable) instances than the ordinary bit-blasting-based decision procedure in Z3. Our method solves roughly the same number of satisfiable and unsatisfiable problems as the default procedure based on bit-blasting in MathSAT, and can handle significantly more satisfiable problems (but fewer unsatisfiable ones) than the ACDCL-based procedure in MathSAT. Few benchmarks are solved by only one solver and they are solved by the best performing solver in their respective category.

**Fig. 5:**
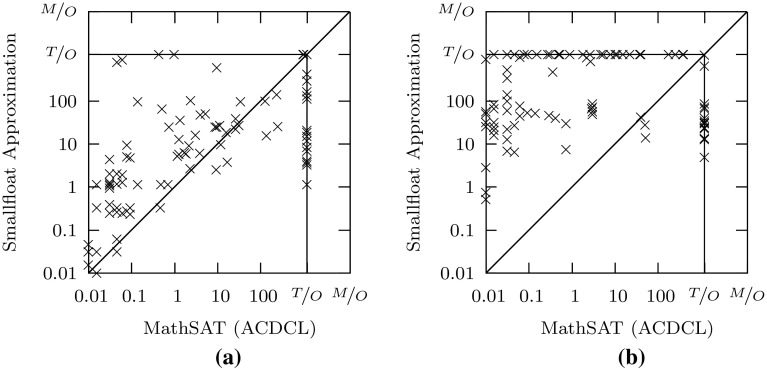
Comparison of our approximation method with the ACDCL-based decision procedure in MathSAT. **a** Satisfiable. **b** Unsatisfiable

Figures 3, 4, 5 provides more detailed results, which show that on satisfiable formulas, our approach (with core-based refinement) is about one order of magnitude faster than Z3, and close to one order of magnitude faster than the default method in MathSAT. In comparison to the ACDCL procedure in MathSAT, the picture is less clear (Fig. 5): while our approximation solves a number of satisfiable problems that are hard for MathSAT, it requires more time than MathSAT on other problems. In addition, the ACDCL procedure outperforms all other methods on unsatisfiable problems.

**Table 4:** Comparison of solver performance on unsatisfiable benchmarks; each entry indicates the number of benchmarks which the approach in the row solves faster than the approach in the column

	Z3 (default)	MathSAT (default)	MathSAT (ACDCL)	Smallfloat (no cores)	Smallfloat
Z3 (default)	–	14	15	59	29
MathSAT (default)	56	–	18	64	52
MathSAT (ACDCL)	73	71	–	75	74
Smallfloat (no cores)	0	5	12	–	2
Smallfloat	35	18	12	62	–

To evaluate the performance of the proof-guided approximation refinement using unsatisfiable cores, we the compare all techniques on the unsatisfiable subset of the benchmarks. Table 4 indicates the numbers of benchmarks on which one approach (the row) performs better (solves vs did not solve, or solves faster) than another approach (the column). Both versions of MathSAT perform much better than the other solvers, which is expected. Of particular interest are the two versions of Smallfloat approximation, since they show the impact of core-based refinement on solving. We can see that Smallfloat, featuring core-based refinement, solves 62 benchmarks faster than Smallfloat (no cores), while it is slower on only two instances. This indicates that core-based refinement offers a substantial improvement over the basic proof-guided refinement. Furthermore, by comparing Smallfloat approximation to Z3 (Default), which is the underlying procedure used by both versions of Smallfloat, we can see that it is faster on 37 instances, whereas Smallfloat (no cores) did not outperform Z3 (Default) on any of the benchmarks. We can conclude that, at least on this benchmark set, the core based refinement offers significant improvement to performance of the approximation framework. It not only improves runtime performance on almost all the benchmarks, it also bridges the gap in performance that is incurred by the approximation framework on more than half of the solved benchmarks.

Overall, it can be observed that our approximation method leads to significant improvements in solver performance, especially where satisfiable formulas are concerned. Our method exhibits complementary performance to the ACDCL procedure in MathSAT; one of the aspects to be investigated in future work is a possible combination of the two methods, using an ACDCL solver to solve the constraints obtained through approximation with our procedure.

## Conclusion

We present a general method for efficient model construction through the use of approximations. By computing a model of a formula interpreted in suitably approximated semantics, followed by reconstruction of a genuine model in the original semantics, scalability of existing decision procedures is improved for complex background theories. Our method uses a refinement procedure to increase the precision of the approximation on demand. Finally, we show that an instantiation of our framework for floating-point arithmetic shows promising results in practice and often outperforms state-of-the-art solvers.

While our prototype exhibits satisfactory performance on unsatisfiable problems, we believe that more work is needed in this area, and that further speed-ups are possible. Furthermore, other background theories need to be investigated, and custom approximation schemes for them be defined. It is also possible to solve approximations with different precision assignments or background theories in parallel, and to use the refinement information from multiple models (or proofs) simultaneously. Increases in precision may then be adjusted based on differences in precision between models, or depending on the runtime required to solve each of the approximations.
